# Lab-on-Paper Devices for Diagnosis of Human Diseases Using Urine Samples—A Review

**DOI:** 10.3390/bios11080260

**Published:** 2021-08-03

**Authors:** Wei-Chun Tai, Yu-Chi Chang, Dean Chou, Lung-Ming Fu

**Affiliations:** 1 Department of Oral and Maxillofacial Surgery, Kaohsiung Chang Gung Memorial Hospital, Kaohsiung 833, Taiwan; ozmt2001@cgmh.org.tw; 2 Department of Engineering Science, National Cheng Kung University, Tainan 701, Taiwan; z10808020@mail.ncku.edu.tw; 3 Department of Biomedical Engineering, National Cheng Kung University, Tainan 701, Taiwan; dean@gs.ncku.edu.tw; 4 Graduate Institute of Materials Engineering, National Pingtung University of Science and Technology, Pingtung 912, Taiwan

**Keywords:** microfluidic, paper-based devices, lab-on-paper, urine, non-invasive samples

## Abstract

In recent years, microfluidic lab-on-paper devices have emerged as a rapid and low-cost alternative to traditional laboratory tests. Additionally, they were widely considered as a promising solution for point-of-care testing (POCT) at home or regions that lack medical infrastructure and resources. This review describes important advances in microfluidic lab-on-paper diagnostics for human health monitoring and disease diagnosis over the past five years. The review commenced by explaining the choice of paper, fabrication methods, and detection techniques to realize microfluidic lab-on-paper devices. Then, the sample pretreatment procedure used to improve the detection performance of lab-on-paper devices was introduced. Furthermore, an in-depth review of lab-on-paper devices for disease measurement based on an analysis of urine samples was presented. The review concludes with the potential challenges that the future development of commercial microfluidic lab-on-paper platforms for human disease detection would face.

## 1. Introduction

Urine is a by-product of kidney metabolism and is rich in many nitrogen-containing substances, including urea, uric acid and creatinine, which are excreted from the body as water-soluble chemicals during urination. The urine volume of normal healthy adults ranges from 0.6 to 2.6 L per day, where approximately 91–96% of this urine is composed of water. However, urine also contains various inorganic salts and organic compounds, such as proteins, hormones, and metabolites. The chemical composition of fresh urine mainly consists of nitrogen, ammonium, ammonia nitrogen, nitrate, nitrite, phosphorus, potassium, sulfate, sodium, magnesium, chloride, and calcium. Moreover, the urine of healthy individuals is clear or light yellow in color. However, in the presence of certain diseases or disorders, such as hematuria, diabetes, or kidney stones, distinct changes in the color, composition or smell of urine may occur. Therefore, urine serves as an important bio-rich resource for health monitoring [1,2].

Unlike blood, urine is a non-invasive sample that can be easily collected without pain, or the need for special equipment. As a result, it has significant potential for point-of-care testing (POCT) or home health monitoring and diagnosis. However, current urinalysis diagnosis techniques still need the use of sophisticated laboratory apparatus and skilled personnel, which precludes their use in the home or in undeveloped areas of the world with poor medical infrastructure and lack of resources. Consequently, lab-on-paper diagnostic platforms have aroused great interest in recent years. Compared to traditional macroscale systems, microfluidic lab-on-paper devices have many advantages, including ease of manufacture, good portability, low cost, a simple diagnostic procedure, and good disposability [3,4,5,6,7,8,9,10]. As a result, they are expected to find increasing use for POCT applications in coming years based on a variety of samples, including urine.

Lab-on-paper devices perform the microanalysis process through patterned microchannels on paper substrates. The flow of the sample and buffer solutions through the device is driven mainly by capillary forces, and hence no external driving source is required. Furthermore, paper is a cheap and easily available material with many options and properties. For example, chromatography paper [11,12,13,14,15] has the advantages of hydrophilicity, cleanliness, homogeneity, reproducibility and biocompatibility, while nitrocellulose (NC) membrane [16,17,18] has a high binding capacity for biomolecules, good stability, and stable reproducibility. Finally, ion-exchange paper and paper towel [19,20] have the advantages of selective separation and permeation, respectively. In fact, the choice of substrate material largely depends on the particular testing requirements.

The fabrication of microchannels or patterns on the substrates of lab-on-paper devices is mainly performed by filling the holes in the paper base with hydrophobic materials to form microchannels and impermeable barriers, or by cutting. The patterning process can be performed using many different methods, including laser printing, inkjet printing, plotting, wax printing, process cutting, flexographic printing, wet etching, laser cutting, screen printing, photolithography, chemical vapor deposition, knife drawing, spray coating, plasma treatment, sol-gel, handheld corona treatment, imprinting, 3D printing, embossing, and so on [21,22,23,24,25,26,27,28]. Each method has its own specific merits and drawbacks. For instance, the screen printing method is capable of producing large-scale devices with a simple process but has a poor hydrophilic-hydrophobic patterning resolution. In addition, the hydrophilic-hydrophobic patterns of paper-based devices made of wax are not suitable for the analysis of organic solvents. As a result, the choice of manufacturing method depends on the specific use and complexity of the device.

Generally speaking, the detection limit and resolution of the lab-on-paper platform depends on the choice of the detection method. Many different detection methods have been adopted, including the fluorescence method, colorimetric method, chemiluminescence (CL) method, electrochemical (EC) method, surface-enhanced Raman spectroscopy (SERS) method, electrochemiluminescence (ECL) method, spectrometry method, and distance-based method [29,30,31,32,33,34,35,36]. Among these methods, distance-based methods and colorimetric methods are the most convenient and do not need the use of any expensive detection apparatus. As a result, they are regarded as particularly promising diagnostic techniques for POCT applications.

This review presents the main advances in the use of urine samples to diagnose human diseases in microfluidic lab-on-paper platforms over the past five years. The article begins by explaining the choice of paper, manufacturing methods, and detection techniques used to implement microfluidic lab-on-paper platforms. Then introduce and explain the sample pretreatment procedure used to improve the performance of lab-on-paper platforms. An in-depth review of recent proposals for microfluidic lab-on-paper platforms for the diagnosis of human diseases using urine samples is then introduced. Finally, the main challenges facing the future development of commercial microfluidic lab-on-paper platforms for urinalysis and diagnosis applications are briefly explored.

## 2. Sample Pretreatment on Paper Devices

The biological sample of the human body is composed of a complex matrix, which is not easy to analyze directly. Therefore, some forms of sample pretreatment procedures were usually needed to extract the target analyte from the matrix to facilitate its downstream detection. Generally, the pretreatment procedure contains various tasks, including sample extraction, storage, separation, collection, and concentration/amplification [37,38]. In recent years, with the rapid improvement of paper-based device fabrication and modification technology, it is now even possible to perform complex sample pretreatment steps on lab-on-paper devices. This section will review in detail some of the recent recommendations for pretreatment of human biological samples on lab-on-paper platforms.

### 2.1. Sample Collection and Storage

**Figure 1:**
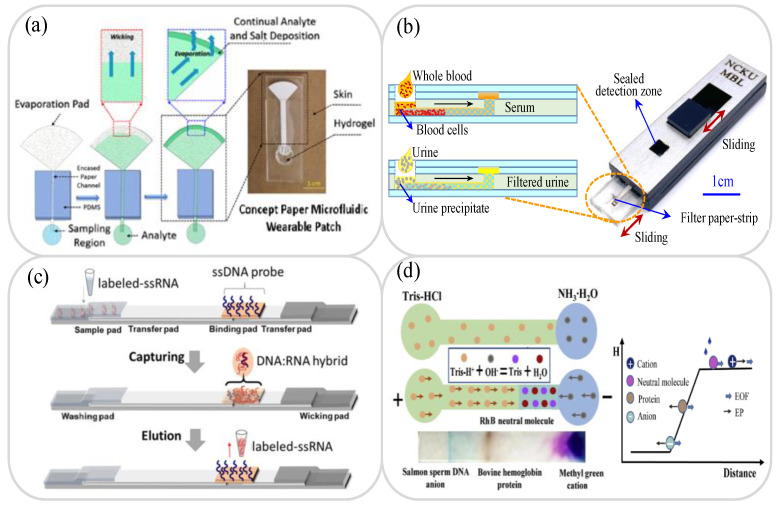
(**a**) Showing working principle of sweat collection in paper-osmotic microfluidic device. Reprinted with permission from ref. [49]. Copyright 2020 AIP Publishing. (**b**) Schematic diagram of PMMA/paper microchip for sample separation. Reprinted with permission from ref. [51]. Copyright 2021 Elsevier. (**c**) Schematic illustration showing working principle of lateral-flow-based extraction of viral RNA on paper device. Reprinted with permission from ref. [63]. Copyright 2020 Elsevier. (**d**) Schematic illustration showing working principle and neutralization reaction of double E/pH gradient concentrator. Reprinted with permission from ref. [84]. Copyright 2020 Elsevier.

In many cases, it is impossible to process and analyze testing samples immediately on collection due to insufficient detection facilities, limited professional technicians, unfavorable on-site conditions, and so on. Consequently, the samples must be stored in some way before being transported to the testing laboratory for and testing and analysis. Traditional sample collection and storage methods usually require complicated procedures and/or large and expensive equipment. Thus, the use of paper chips, suitably modified through surface treatments such as nitrification and/or the addition of appropriate surfactants has attracted growing interest as an alternative sample collection and storage technology in recent years [39,40]. For example, fast technical analysis (FTA) cards have now been successfully commercialized for the collection and storage of blood samples, urine samples, plasmids, cells, and viruses [41,42,43,44]. In addition, many lab-on-paper platforms have also been developed for the collection and storage of blood, urine, saliva, sweat and tear samples [45,46,47,48,49]. For instance, Shay et al. developed a wearable lab-on-paper platform with a capillary–evaporative transport function for the collection of sweat samples for long-term measurement and analysis [49]. As shown in Figure 1a, typical paper-based sample collection and storage devices comprise three parts, namely a region for storing the sample fluid, a closed paper channel for transporting the sample fluid, and a paper pad with a large surface area for driving the sample fluid continuously through the device under the effects of evaporation.

### 2.2. Sample Separation

For many lab-on-paper applications, it is necessary to pre-treat the raw sample prior to analysis using separation, purification and extraction methods, in order to minimize the effects of interference errors and possible diagnostic distortion. For example, whole blood samples comprise a mixture of red blood cells (RBCs), white blood cells (WBCs), platelets, and plasma, and if the plasma is not properly separated from the whole blood, the determination of the detection signal during the detection process may be severely impaired. Accordingly, many lab-on-paper platforms for the separation of whole blood samples have been proposed in recent years. Generally speaking, these devices perform the separation process using filter paper [50,51], filtration membranes [52,53,54,55], chitosan polymer structure separation [56], or combined dielectrophoretic (DEP) and capillary force separation [57]. For example, Laurenciano et al. [51] fabricated a sliding hybrid PMMA/paper microchip with an integrated separation membrane to filter urine samples or separate whole blood (Figure 1b). The feasibility of the proposed device was demonstrated by filtering human urine samples to measure the concentration of total protein (TP) and separating whole blood samples to measure the concentrations of albumin (ALB) and creatine (CRE), respectively.

### 2.3. Sample Extraction

In the detection process, the extraction step always requires separation of the target analyte (e.g., cells, virus, ions, proteins, DNA, drugs, RNA, and so on) from the collected sample (e.g., biological, environmental, or food) for subsequent measurement and analysis. Many paper devices have been proposed for sample extraction [58,59,60,61,62,63,64,65,66,67,68], including commercial FTA and FTA elute cards [41,43]. However, while these cards are easy to use, their integration with lab-on-paper platforms still presents a significant challenge. Accordingly, many studies have attempted to implement the extraction function on the paper platform itself extraction device of lab-on-paper can be used for DNA, RNA, dug, heavy metal ions, and pesticide residues, and so on [61,62,63,64,65,66,67,68]. For instance, Batule et al. [63] presented a lab-on-paper platform based on nucleic acid testing for the rapid extraction and detection of virus RNA from blood serum samples. The device consisted of an assembly of seven pads mounted on a backing card, namely a washing pad, three transfer pads, a sample pad, a binding pad and a wicking pad (see Figure 1c). The binding pad was modified by DNA probes designed to capture SS virus RNA, and the RNA extraction process involved a lysis of the virus particles, followed by hybridization and elution. It was shown that the proposed platform successfully extracted the RNA from various viruses, including Zika virus, dengue fever and chikungunya fever, within 5 min.

### 2.4. Sample Concentration/Amplification

To enhance the detection limit of paper-based platforms, it is frequently necessary to perform some form of sample preconcentration or amplification procedure prior to the analysis and detection process. Sample pre-concentration methods typically employ ion concentration polarization (ICP) [69,70], isotachophoresis (ITP) [71,72], electrokinetic stacking (EKS) [73,74], or multiplex electroanalytical techniques [75]. Meanwhile, sample amplification methods generally utilize loop-mediated isothermal amplification (LAMP) [76,77,78,79,80], catalytic hairpin assembly (CHA) amplification [81,82], or hybrid chain reaction (HCR) [82,83]. Cai et al. [84] presented a lab-on-paper device for the preconcentration of urine microalbuminuria (MAU) proteins by the coalesced effects of a pH gradient and an electric field. The proposed device achieved a 100-fold amplification of ALB in artificial urine medium within 70 s (see Figure 1d). Moreover, the detection results obtained for the MAU content of urine samples collected from real diabetic patients were found to be in excellent agreement with those obtained using a traditional immunoturbidimetry method.

## 3. Application of Lab-on-Paper Platforms to Human Disease Diagnosis Using Urine Samples

Urine samples are easy to obtain and have particular biological richness (including urea, amino acids, chlorides, potassium, phosphates, sodium, sulfates and other trace chemical elements). A comparison of detection using human samples is shown in Table 1. Furthermore, the average person produces a large quantity of urine every day (400 to 2000 mL). Consequently, urine represents an ideal non-invasive sample for biomarker detection purposes [85]. For example, abnormal quantities of urinary oxalate and calcium can provide an indication of kidney stones [86], while excessive quantities of urinary glucose and protein may be a sign of diabetes [87]. Similarly, high levels of urinary serotonin can be an early indicator of melancholia [88]. The literature thus contains many proposals for lab-on-paper platforms based on urine samples for the detection of diabetes, kidney disease, liver and heart function disorders, pregnancy, drugs, heavy metals, and so on.

### 3.1. Glucose Analysis

**Figure 2:**
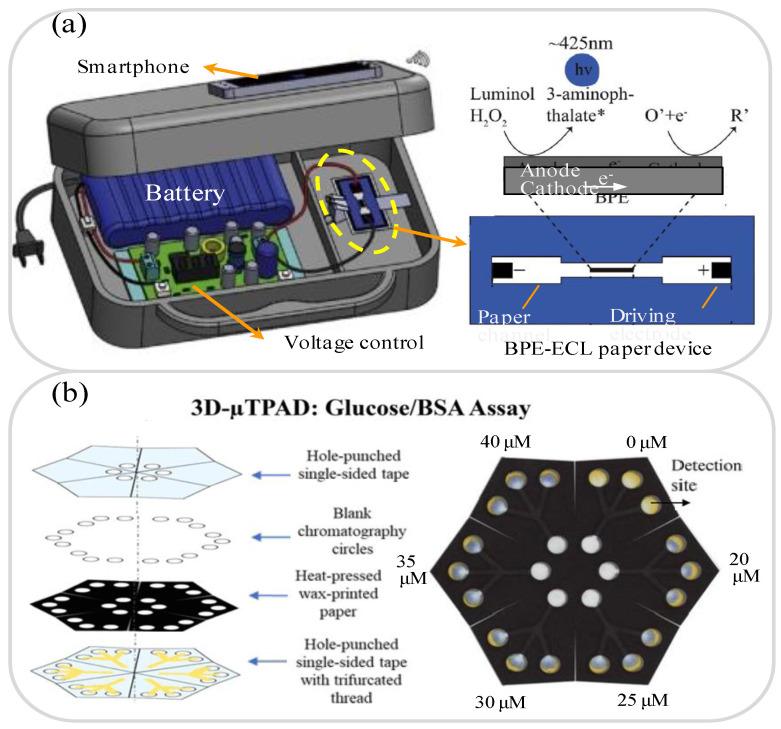
(**a**) P-BPE-ECL system and BPE-ECL paper device. Reprinted with permission from ref. [93]. Copyright 2016 Elsevier. (**b**). 3D-μTPAD platform with colorimetric detection for glucose in artificial urine samples. Reprinted with permission from ref. [103]. Copyright 2019 Wiley.

Diabetes is a chronic metabolic abnormality characterized by elevated blood sugar levels over a long period of time. When the blood sugar level exceeds 180 mg/dL, glucose appears in the urine, causing a so-called glucose in urine (GLU) disorder [89,90]. GLU is easily detected through simple urine tests and is regarded as a reliable indicator of early diabetes and kidney disease [91]. Li et al. [92] developed a paper-based electrochemical device (PED) for the detection of glucose in artificial urine, in which the carbon working electrode was patterned using a pressure-assisted ballpoint pen and the silver reference electrode was deposited directly on the paper. The PED was used as a test strip in combination with a commercial blood glucometer and was found to achieve a linear glucose detection range of 2.0 to 20 mM with a LOD of 2.0 mM. Chen et al. [93] developed a handheld paper-based bipolar electrode-electrochemiluminescence (P-BPE-ECL) chip for the detection of glucose in phosphate-buffered saline (PBS) solution and artificial urine samples. As shown in Figure 2a, the BPE incorporated an electronic conductor between the anode and the cathode and produced oxidation and reduction reactions under the effects of an external DC voltage when immersed in electrolyte solution. In the proposed detection method, the glucose level was determined by the P-BPE-ECL assay. The experimental results showed that the P-BPE-ECL system achieved LODs of 0.017 mM and 0.030 mM in PBS and artificial urine, respectively. In other recent studies [87,94,95,96] lab-on-paper platforms were integrated with EC or ECL sensors to perform glucose detection in urine with LODs ranging from 0.35 mM to 3 × 10^−5^ mM.

Many lab-on-paper platforms with integrated colorimetric detection [97,98,99,100,101] have been proposed for determining the glucose concentration in human urine samples. de Oliveira et al. [102] developed 2D and 3D lab-on-paper platforms for the integrated colorimetric detection of glucose, total protein and nitrite in serum and artificial urine. The two lab-on-paper platforms were manufactured using a paper cutter printer and contained separate detection areas for the colorimetric detection of the three analytes of interest. The results obtained using artificial urine samples, showed that the 2D lab-on-paper platform achieved LODs of 0.54 mM, 5.19 μM and 2.34 μM for glucose, protein and nitrite, respectively. The corresponding LODs of 3D lab-on-paper platform used to detect glucose, protein and nitrite in artificial serum were 0.44 mM, 1.26 μM and 4.35 μM. Neris et al. [103] presented a 3D lab-on-paper device and multilayer microfluidic line/paper-based analysis device (3D-μTPAD) for the colorimetric detection of glucose in artificial urine samples. The 3D lab-on-paper device comprised three layers, i.e., wax, hot-pressed wax printing paper and single-sided tape, while the 3D-μTPAD consisted of four layers, namely a hole-punched layer, a chromatography layer, a heat-pressed wax-printed paper layer, and a hole-punched layer containing trifurcated thread (see Figure 2b). Glucose oxidase solution was flowed through each layer; resulting in the formation of a yellow-brown reaction complex following contact with glucose. The 3D lab-on-paper device and 3D-μTPAD devices achieved LODs of 1.0 mM and 0.5 mM, respectively, when applied to artificial urine samples. Table 2 briefly reviews several other lab-on-paper platforms presented in recent studies for the detection of glucose in urine samples.

### 3.2. Creatinine, Albumin, Protein and Uric Acid Analysis

Chronic kidney disease (CKD) usually has no obvious symptoms during the early development stage, and hence often passes undetected until finally diagnosed. As a result, the effective treatment period is curtailed, leading to serious lesions and a significant deterioration in quality of life [104]. However, recent research has shown that lab-on-paper platforms provide a feasible means of detecting early-stage kidney disease by evaluating the concentrations of creatinine (Cre) and albumin (Alb) [105,106,107,108,109,110,111], micro-albumin and protein [112,113,114,115,116], and uric acid [117,118,119,120,121,122,123] in urine samples.

**Figure 3:**
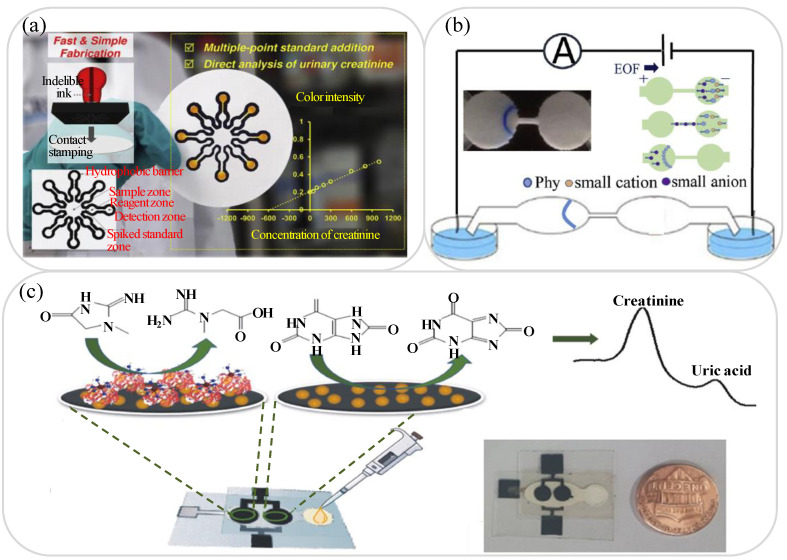
(**a**) Lab-on-paper device fabricated by contact stamping for multi-point standard addition analysis. Reprinted with permission from ref. [108]. Copyright 2020 Elsevier. (**b**) Lab-on-paper device integrated with field-amplified stacking technology and desalination during stacking process. Reprinted with permission from ref. [112]. Copyright 2019 Elsevier. (**c**) Schematic illustration showing working principle of lab-on-paper EC sensor device for the detection of Cre and uric acid. Reprinted with permission from ref. [122]. Copyright 2019 Elsevier.

Healthy kidneys play a vital role in maintaining human health by removing waste and excess fluid from the blood and expelling them with extra water in the form of urine. An excess quantity of creatinine (Cre) in the urine is a reliable indicator of early-stage kidney disease [105]. Thus, in recent years, various lab-on-paper devices have been proposed for Cre detection in urine samples [106,107]. For instance, Sununta et al. [106] demonstrated a colorimetric lab-on-paper device based on the Jaffé reaction of Cre with picric acid under alkaline conditions. The intensity of the resulting orange color Cre-alkaline picrate complex was analyzed using ImageJ software to detect the Cre concentration. The device achieved a wide linear detection range of 0.2 to 1 mM with limits of detection and quantitation of 0.08 and 0.26 mM, respectively. Mathaweesansurn et al. [108] presented a contact-imprinted lab-on-paper device for the multi-point determination of Cre in human urine. As shown in Figure 3a, a single-step embossing method was first performed using a rubber stamp to fabricate eight individual hydrophobic barrier structure patterns on the lab-on-paper platform for multi-point standard analysis. As in the device proposed by Sununta et al. [106], Cre detection was performed colorimetrically by inducing a Jaffé reaction between the Cre content in the urine sample and the picric acid embedded in the reaction zones of the lab-on-paper device. The device was shown to have a linear Cre detection range of 50 to 1000 mg/L and a LOQ of 16.9 mg/L. The urine Alb to Cre ratio can also be used as a screening strategy for kidney disease [109]. Hiraoka et al. [110] developed a Drawing lab-on-paper device for urinary Alb-to-Cre ratio analysis to support the early detection of renal insufficiency. In the proposed device, the Alb-to-Cre ratio was determined simply by drawing a straight line between the top of the two color-changed assay channel sections (Alb and Cre) and then observing the position of the intercept point on an Alb-to-Cre scale placed between them. The results obtained for 12 urine samples collected from a hospital showed that the detection results for the Alb-to-Cre ratio were within 15% of the theoretical values.

Micro-albumin and urinary protein measurement also provide a feasible means of performing the early-stage detection of kidney disease [112,113,114]. Cai et al. [112] presented a field-amplification lab-on-paper device for the sensitive colorimetric detection of micro-albuminuria (MAU) in human urine. As shown in Figure 3b, the geometric shape of a 2D paper-based microfluidic channel was used to introduce an electric field gradient and achieve a field amplification effect by progressively reducing the sample conductivity. In the detection process, albumin (colorless) was selectively stained with bromophenol blue (BPB), and after passing through the stacking band of the lab-on-paper device, the MAU intensity was analyzed using ImageJ software and the corresponding micro-albumin concentration was determined. The experimental results showed that the device achieved a linear MAU concentration range of 10 to 100 mg/L with a LOD of 6.5 mg/L. Gao et al. [115] proposed a lab-on-paper platform incorporating a cation exchange membrane for the ion concentration polarization (ICP) stacking and colorimetric detection of total protein in urine. It was shown that the ICP stacking effect enhanced the sensitivity of the proposed device by about 60 times. Moreover, the linear detection ranges of human serum albumin (HSA) extended from 50 to 350 mg/L, while the recovery rate was 93 to 108% compared to the total protein detected in clinical urine samples. The same group [84] later demonstrated a second lab-on-paper platform for HSA concentration determination, in which the detection sensitivity was enhanced by a factor of around 100 times by purifying and enriching the protein content under a combined electric field and pH gradient. The device showed a linear HSA detection range of 10 to 300 mg/L in artificial urine samples and a LOD of 4.9 mg/L.

Uric acid is a heterocyclic consisted of oxygen, nitrogen, carbon and hydrogen, and is a natural product of the metabolic breakdown of purine nucleotides in the human body. A high concentration of uric acid in urine is not only a forewarning of gout [116,117], but also a strong indicator of kidney disease [118] as well as diabetes, hyperuricemia and urolithiasis [119]. Thus, many lab-on-paper platforms for the detection of uric acid in urine samples have been proposed in recent years [117,118,119,120,121,122,123]. Ali et al. [119] demonstrated a colorimetric lab-on-paper device for the quantitative detection of uric acid using citrate-terminated Pt nanoparticles (PtNPs) as sensing probes. The PtNPs served as a catalyst in producing an oxidation effect, which was subsequently reduced in the presence of uric acid, resulting in a detectable color change from blue to yellow. Compared to the detection results obtained using a standard colorimetric technique without the citrate-capped PtNPs, the proposed device showed a wider linear detection range of 0 to 8 mM and a LOD almost one order higher. Villa and Poppi [120] developed a quantitative method for detecting uric acid in urine using a surface-enhanced Raman spectroscopy (SERS) technique in combination with a lab-on-paper device coated with AuNPs. The device had a linear detection range of 0 to 3.5 mM (mmol/L) and a LOD of 0.11 mM. Several studies have attempted to increase the sensitivity of the uric acid concentration detection process by integrating lab-on-paper platforms with EC sensors [121,122]. For example, Cincotto et al. [122] demonstrated a simple disposable lab-on-paper device consisting of two EC sensors for the simultaneous determination of uric acid and Cre, respectively, in human urine samples. In the proposed platform, graphene quantum dots (QDs) were used to enhance the oxidation of the uric acid and Cre on the working electrode and the concentrations of the two analytes were then determined via EC analysis with square-wave voltammetry (see Figure 3c). The results showed that the platform was capable of detecting uric acid and creatinine concentrations in the range of 0.010 to 3.0 μM (μmol/L) with LODs of 8.4 nM (nmol/L) and 3.7 nM, respectively. Table 2 briefly reviews several other lab-on-paper platforms presented in recent studies for the detection of kidney disease using urine samples.

**Table 2:** Summarizes the μPADs used for urine analysis.

Ref.	Materials and Structures	Fabrication Methods	Detection Methods	Target and Sample Matrices	Detection Range	Detection Limit
[87]	Filter paper, 3-D	Wax printing + screen printing	EC	Glucose	0.1–40 mM	0.03 mM
[89]	Filter paper, 3-D	Wax printing + screen printing	EC	Glucose	0.5–5 mM	0.5 mM
[90]	Filter paper, 2-D	Laser cutting	CM	Glucose	0–150 μM	2.5 μM
[95]	Filter paper, 3-D	Wax printing + screen printing	EC	Glucose Creatinine Uric acid	0–5 mM	0.12 μM 0.084 μM 0.012μM
[96]	Filter paper, 2-D	Cutting + coating	EC	Glucose	0.1–1 mM	25 μM
[97]	Filter paper, 2-D	Cutting	CM	Glucose	0–56 mM	0.54 mM
[98]	Thread + Chromatography paper, 3-D	Wax printing + cutting	CM	Glucose	0–15 mM	0.5 mM
[99]	Chromatography paper, 2-D	Inkjet printing	CM	Glucose	0.01–4 mg/mL	0.01 mg/mL
[100]	Filter paper, 3-D	Spray + punch	CM	Glucose Nitrite	0.05–0.7 mM 0.02–0.9 mM	25 μM 48 μM
[105]	Filter paper, 2-D	Cutting	CM	Creatinine	10–60 mg/L	4.2 mg/L
[107]	Filter paper, 2-D	Cutting	ISE	Sodium Potassium Creatinine	0–36.99 mM 0–7.78 mM 0–15.65 mg/dL	0.95 mM 0.47 mM 0.59 mg/dL
[117]	Filter paper, 2-D	Wax printing	CM	Creatinine Uric acid	50–600 mg/L 50–500 mg/L	15.7 mg/L 16.5 mg/L
[121]	Filter paper, 3-D	Inkjet printing + screen printing	EC	Dopamine Uric acid	20–1000 μM	2.19 μM 1.80 μM

CM: colorimetric; EC: electrochemical; ISE: selective electrode method.

### 3.3. Bacteria and Cancer Analysis

**Figure 4:**
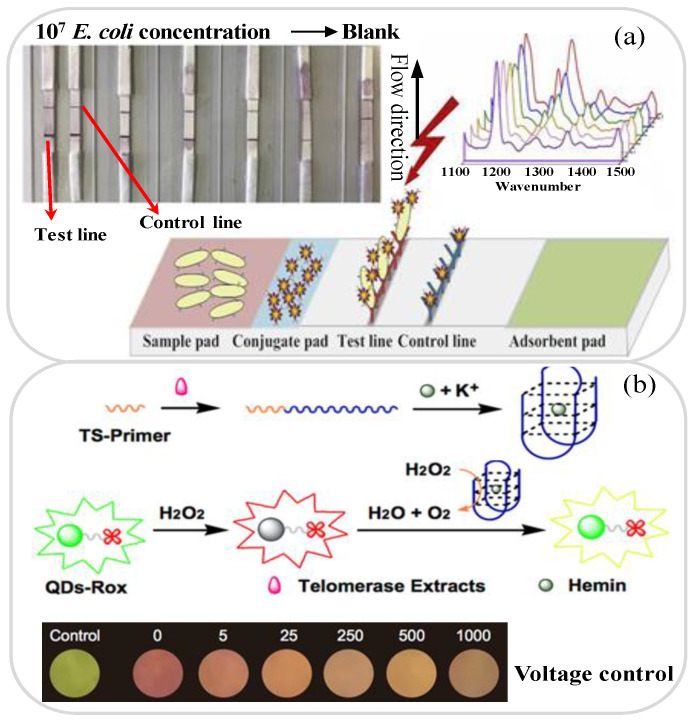
(**a**) μPAD-LFIA platform with SERS detection-coupled immunomagnetic enrichment for *E. coli* detection. Reprinted with permission from ref. [126]. Copyright 2019 Elsevier. (**b**) Lab-on-paper device with fluorescent probes for telomerase activity assay in urine for bladder cancer diagnosis. Reprinted with permission from ref. [132]. Copyright 2019 Elsevier.

Healthy urine contains no bacteria. However, bacteria in the anus can easily retrograde to the bladder through the urethra. For normal healthy individuals, the bladder can remove these bacteria on its own. However, in some cases, the bacteria may remain in the urinary system and cause infection [124,125]. In fact, most urinary tract infections (UTI) are caused by Escherichia coli (*E. coli*) [126,127,128], which normally appears in the rectum. Consequently, several lab-on-paper platforms aimed at the detection of *E. coli* in urine have been proposed in recent years [125,126,127,128,129]. For example, Gumustas et al. [125] proposed a paper-based test strip for the sensitive detection and quantification of *E. coli* in urine samples by means of SERS-based and colorimetric lateral flow immunoassay (LFIA) techniques. In developing the strip, the detection sensitivity was improved by using AuNPs modified with Raman labels to enhance the SERS response. The results showed that the colorimetric and SERS methods achieved LOD values of 78 and 45 CFU/mL, respectively. Ilhan et al. [126] presented a lab-on-paper platform with SERS detection-coupled immunomagnetic enrichment for the detection of *E. coli* in urine samples. As shown in Figure 4a. Fe_3_O_4_@Au nanoparticles combined with E. coli antibodies were used for selective separation and enrichment, and the casein-modified Fe_3_O_4_/Au-PEI nanoparticles were then cleaved; enabling the SERS detection process to achieve a more sensitive quantitative analysis. The platform achieved a linear *E. coli* detection range of 101 to 107 CFU/mL and a LOD of 0.52 CFU/mL. Calabretta et al. [128] developed a bioluminescent sensing lab-on-paper platform based on a luciferase/D-luciferin reaction for detecting bacterial adenosine-5′-triphosphate (ATP) for UTI diagnostics. The proposed platform used an innovative freeze-drying procedure and stable nano-lantern structured ATP-sensing paper biosensor for the effective detection of *E. coli* cells in urine samples within 10 min. In experimental tests, the device showed a LOD of 10^−14^ mol ATP (corresponding to 3.8 × 10^4^ CFU/mL).

Lab-on-paper platforms can also be used to detect many different types of cancer cells in urine, including Hela cells, bladder cancer cells, prostate cancer cells and microRNA-21 [130,131,132,133,134]. Zhang et al. [131] presented a fluorescence detection method based on polyacrylamide dendrimer-activated chromatography-based paper for the detection of telomerase activity in HeLa cell lysates. The paper platform was manufactured using a manual drawing technique and exploited the hybridization of Cy5-modified single-stranded DNA probes with telomerase extension products for the detection of telomerase activity. The results show that the platform can detect telomerase activity in HeLa cell lysates of only 10 cells. Ma et al. [132] demonstrated a lab-on-paper based on Rox-DNA functionalized CdZnTeS quantum dots (QDs) with fluorescent probes to detect bladder cancer cells in urine samples. As shown in Figure 4b, the Rox-DNA functionalized QDs were first quenched by H_2_O_2_ and then catalyzed as G-quadruplex reactants; thereby minimizing the interference of the H_2_O_2_ on the green fluorescence emissions. The LOD and response time were shown to be 10 cells and less than 60 min, respectively. Abarghoei et al. [133] presented a colorimetric lab-on-paper sensor based on the peroxidase-like activity of cysteine-capped gold nanoclusters (CysAuNCs) for citrate detection in patients with prostate cancer. The Cys-AuNCs had an inherent peroxidase mimicking activity, and hence served both to promote catalytic oxidation and generate blue dye. As a result, they provided the means to measure the citrate ions directly without modification. The proposed sensor showed a good linear response over the citrate concentration range of 1.0 μM to 10 mM and achieved a LOD of 0.4 μM. Table 3 briefly reviews several other lab-on-paper platforms presented in recent studies for the detection of bacteria and cancer cells in urine samples.

### 3.4. Drugs and Ion Analysis

**Figure 5:**
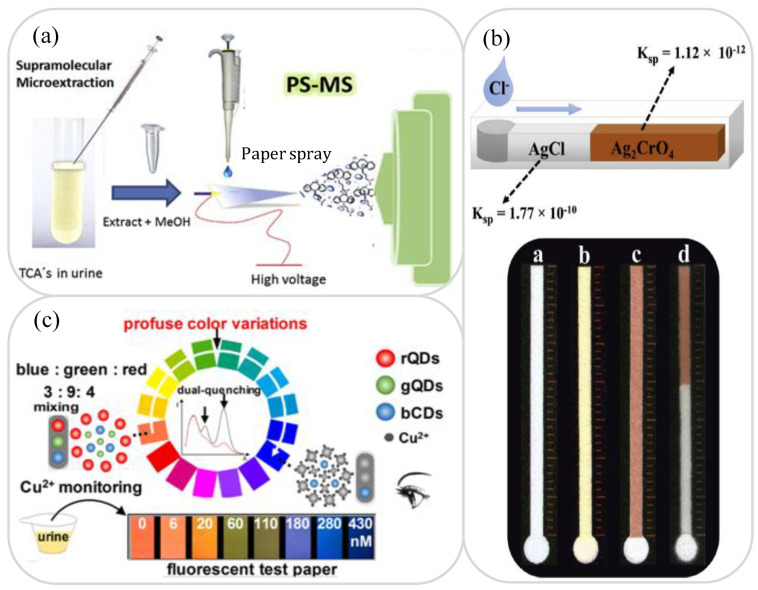
(**a**) Paper spray ionization mass spectrometry detection device for sensitive determination of tricyclic antidepressants in urine. Reprinted with permission from ref. [137]. Copyright 2020 Elsevier. (**b**) Lab-on-paper device based on argentometric distance-based for the detection of chloride ion concentration. Reprinted with permission from ref. [143]. Copyright 2019 Elsevier. (**c**) Lab-on-paper device with fluorescent sensor for visual detection of Cu^2+^ using three-color probe. Reprinted with permission from ref. [146]. Copyright 2018 Elsevier.

In general, the term “drug” refers to any substance that causes physiological or psychological changes in an organism when used. Moreover, “prohibited drugs” are psychotropic or narcotic drugs that are used for recreational “non-medical purposes” and may cause dependency and eventual addiction. Many lab-on-paper platforms have been proposed for the detection of drugs such as caffeine, paracetamol, adenosine, cocaine, ketamine, cannabidiol (CBD), methamphetamine (MA), 3,4-methylenedioxy -N-methamphetamine (MDMA) and tricyclic antidepressants [135,136,137,138,139,140,141,142]. Petroni et al. [135] demonstrated a lab-on-paper platform with a CE sensor coupled to an external graphene-Cu nanoparticle-modified solid electrode through a meniscus configuration for the detection of paracetamol and caffeine in urine samples. The working electrode had the form of a glassy carbon electrode consisting of graphene oxide-copper nanoparticles (GO-CuNPs) synthesized via the chemical reduction of GO and Cu(II) salt. The device was shown to be capable of performing the quantitative analysis of paracetamol and caffeine in real urine samples with LODs of 24.6 nM and 36.1 nM, respectively. de Oliveira et al. [137] presented an electric paper-based spray (PS) platform combined with ionization mass spectrometry (MS) for the supramolecular microextraction and sensitive determination of four antidepressants (amitriptyline, doxepin, imipramine and nortriptyline) in urine (see Figure 5a). The supramolecular microextraction process increased the pre-concentration factor of the tricyclic antidepressants in the urine sample and improved the sensitivity of the detection process as a result. The proposed platform achieved a LOD and LOQ of 5.2 to 8.6 μg/L and 7.4 to 28.7 μg/L, respectively. The literature contains various other PS-MS platforms for the rapid and sensitive analysis of psychiatric or anesthetic drugs in urine [138,139,140]. Narang et al. [141] developed an ultrasensitive technique for ketamine detection in three samples (whiskey, urine and fruit juice) using a zeolite nanocrystal-modified lab-on-paper platform coupled with GO nanosheets and an EC sensor. The lab-on-paper platform had a linear range of 0.001 to 5 nM/mL for the detection of ketamine in the three different samples and achieved a LOD of 0.001 nM/mL. Moreover, when applied to real-world samples such as alcohol and non-alcoholic beverages, the correlation between the detection results and the ground truth results was found to be as high as 99%.

The ion content and composition in human urine provide many useful insights into the health of the individual. For example, excessive sodium content is often associated with disorders such as blood pressure and cardiovascular disease, while a high potassium content may indicate low blood pressure and arrhythmia [107]. Similarly, a high chlorine concentration is frequently associated with kidney function disorders [143]. Heavy metal ions, such as Cu^2+^, Pb^+^ and Au^3+^, are easily deposited in the body through foods and drinks and cause chronic poisoning when present in excessive quantities. Accordingly, the problem of detecting electrolytes and heavy metal ions in human urine samples using lab-on-paper platforms has attracted significant attention in recent years [144,145,146,147,148,149,150]. Rahbar et al. [143] proposed a trapezoidal distance-based lab-on-paper platform with reverse Mohr precipitation titration for the determination of microscale quantities of chloride in urine samples (see Figure 5b). In the proposed device, Ag_2_CrO_4_ crystals were initially formed along the microfluidic detection channel and when the Cl^−^ was then introduced at the microchannel inlet, the Cl^−^ progressively replaced the CrO_4_^2−^ in the complex and formed white AgCl crystals in its place. The length of the AgCl crystal band along the microchannel was then used to measure the concentration of chloride within the sample. It was shown that for a microchannel with the same area and length, the sensitivity achieved using a trapezoidal ascent channel (i.e., a diffusion type channel) was better than that achieved using a rectangular and descending trapezoid channel (i.e., a tapered type channel). When using a channel of the former type, the device achieved a linear concentration range of 0.05 to 25 mM and a LOD of 0.05 mM. Cai et al. [146] presented a color-evolution-based lab-on-paper platform with a tricolorratiometric fluorescent sensor for the visual detection of endogenous Cu^2+^ ions in human urine. As shown in Figure 5c, the three-color probe sensor comprised blue-emission carbon dots (bCDs), red-emissive QDs (rQDs) and green-emissive QDs (gQDs). The fluorescence of the gQDs and rQDs was quenched in the presence of Cu^2+^. However, the fluorescence of the bCDs was unaffected by the Cu^2+^ ions, and hence served as an internal standard for the light stability. It was shown that the color of the probe gradually changed from light pink to dark blue as the Cu^2+^ concentration increased. The device presented a linear detection range for Cu^2+^ ions in urine samples of 3 to 430 nM and a LOD of just 1.3 nM. Faham et al. [149] demonstrated a lab-on-paper colorimetric platform with an optical probe based on AuNPs modified with 2,2′-thiodiacetic acid (TDA) for the determination of Cr(III) and Cr(VI) ions in urine and diluted human plasma samples. The Cr(III) ions induced the aggregation of the modified AuNPs and prompted a color change of the TDA-AuNPs probe from red to blue, which was subsequently detected using a colorimetry technique implemented on a smartphone. Meanwhile, the Cr(VI) concentration was inferred by reducing the concentration of the Cr(III) ions using ascorbic acid and then quantifying the total Cr(III) concentration and subtracting this value from the total chromium concentration. The lab-on-paper platform had a linear detection range of 1.0 nM to 1.0 mM for Cr(III) and a LOD of 0.64 nM. Table 3 briefly reviews several other lab-on-paper platforms presented in the recent literature for drug detection and ion analysis in urine samples.

### 3.5. Other Analytes

**Figure 6:**
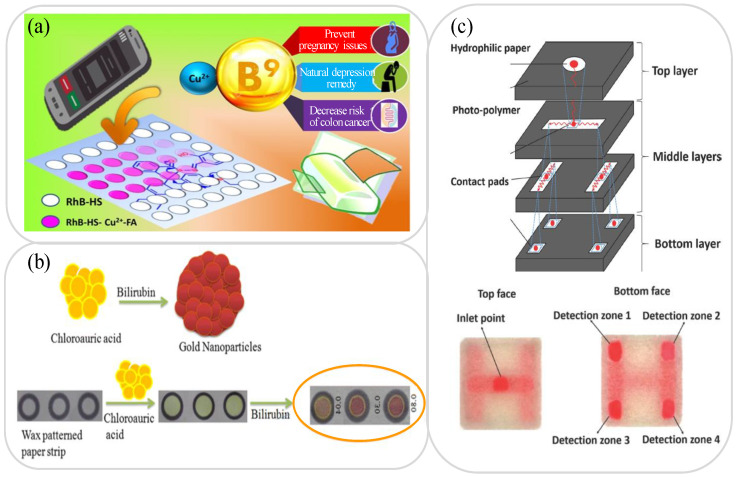
(**a**) Lab-on-paper platform with colorimetric probe for FA detection based on open-ring form amplification of RhB derivative. Reprinted with permission from ref. [154]. Copyright 2020 Elsevier. (**b**) Lab-on-paper device with in-situ formation of AuNPs for colorimetric determination of bilirubin. Reprinted with permission from ref. [156]. Copyright 2020 Springer. (**c**) A stacked four-layer cellulose paper lab-on-paper device for detection of BSA, glucose, nitrite and pH value in urine sample. Reprinted with permission from ref. [101]. Copyright 2020 Elsevier.

In addition to the lab-on-paper platforms described above, paper-based devices have been proposed for the detection of many other analytes in urine samples, including serotonin (symptoms: regulating physiological processes) [151,152], folic acid (also known as vitamin B9; symptoms: physical weakness, irritability, anemia and leucopenia) [153,154], urobilinogen and billirubin (symptoms: liver disease, jaundice, hemolysis) [155,156], 8-hydroxy-2-deoxyguanosine (8-OHdG; symptoms: aging, diabetes, inflammation) [157,158], and phenylketonuria (PKU; symptoms: metabolic diseases, delayed growth and development). Amatatongchai et al. [151] demonstrated a 3D folded lab-on-paper platform for the highly sensitive detection of serotonin in urine samples using a three-electrode EC sensor consisting of a working electrode (WE), a reference electrode (RE), and a counter electrode (CE). The WE was modified with Fe_3_O_4_@Au@SiO_2_ and coated with molecularly imprinted polymers (MIP) in order to improve its selectivity for serotonin. The results showed that the device had a linear serotonin detection range of 0.01 to 1000 μM under the anodic peak current and a LOD of 0.002 μM. Nghia et al. [154] developed a lab-on-paper platform with colorimetric probes for the detection of folic acid (FA) in serum and urine samples. As shown in Figure 6a, the FA probe exploited the enhanced transfer of electrons in the presence of FA from the rhodamine B (RhB) derivative to Cu^2+^ during the ring-opening amplification of the RhB derivative. In particular, after the addition of FA, the colorless spirolactam ring was linked by the Cu^2+^ ions into a ring form and turned pink under the structural transformation of the RhB derivative. The probe showed good linearity over the FA concentration range of 40 to 260 nM with a LOD of 22 nM.

Edachana et al. [156] demonstrated a lab-on-paper device based on colorimetric method with AuNPs for the detection of bilirubin in urine samples. When chloroauric acid was added to the detection zone, the resulting yellow Au(III) complex reduced to purple AuNPs in the presence of bilirubin, and the color change was used to analyze the bilirubin concentration (see Figure 6b). The intensity of the purple AuNPs increased linearly with an increasing bilirubin concentration in the range of 5.0 to 1000 mg/L. Moreover, the detection limit was 1.0 mg/L. Meng et al. [157] employed a 3-film paper-based solid-phase microextraction (p-SPME) device for the analysis of 8-OHdG in urine samples using a capillary electrophoresis laser-induced fluorescence detection (CE-LIF) technique. The p-SPME device leveraged the large surface area of the cellulose molecules and their preferential bonding with hydrogen to enhance the concentration of the 8-OHdG sample and minimize the interference of the CE-LIF separation effect. The device had a linear detection range of 10 to 1000 nM and a LOD of 5 nM. Messina et al. [159] demonstrated a lab-on-paper platform to quantify the concentration of phenylalanine in patients with PKU from urine samples. In preparing the device, phenylalanine ammonia lyase (PAL) enzymes and reagents were immobilized on the surface matrix of the lab-on-paper platform and when PKU was subsequently added, it reacted with the PAL and reagents under temperature control to produce a detectable change in the color intensity. The device exhibited a linear response over the range of 20 μM to 3000 μM and achieved a LOD of 20 μM. In addition, the detection of pH in urine is also very important, and it is also one of the main indicators for determining urine detection [160]. Galanis et al. [101] developed a stacked four-layer cellulose paper and made a 3D multi-layer lab-on-paper device (see Figure 6c). The lab-on-paper device can detect BSA, glucose, nitrite and the pH value of test samples mixed in artificial urine. The pH value test uses bromothymol blue as the indicator, dilutes the red chemical dye with deionized water, and the detectable range is 6.0~7.6. Table 3 briefly reviews several other lab-on-paper platforms presented in the literature for the detection of various other analytes in urine samples.

**Table 3:** Summarizes the μPADs used for urine analysis.

Ref.	Materials and Structures	Fabrication Methods	Detection Methods	Target and Sample Matrices	Detection Range	Detection Limit
[126]	Stripe paper, 3-D	Cutting + bonding	SERS	*E. coil*	10^1^–10^7^ CFU/mL	0.52 CFU/mL
[128]	Filter paper, 3-D	Wax printing + cutting	CM	Bacterial ATP	3.3 × 10^−9^–3.3 × 10^−15^ mol	3.8 × 10^−14^ mol
[130]	Filter paper, 2-D	Deposition	SERS	Adenosine	3.8–4.9 μM	3.8 μM
[134]	Filter paper, 2-D	Laser cutting	CM	miRNA-21	10–1000 pM	4.1 pM
[138]	Chromatography paper, 2-D	Cutting	PS-MS	Caffeine CBD CBN Cocaine	10–100 ng/mL	7.8 ng/mL 11.4 ng/mL 1.4 ng/mL 2.1 ng/mL
[140]	Filter paper, 2-D	Cutting	MS	Indomethacin Diclofenac Tolmetin Ketoprofen Naproxen Ibuprofen	25–1000 μg/L 25–1000 μg/L 25–1000 μg/L 50–1000 μg/L 50–1000 μg/L 50–1000 μg/L	3.8 μg/L 7.2 μg/L 6.8 μg/L 9.4 μg/L 15.7 μg/L 5.1 μg/L
[142]	Filter paper, 2-D	Cutting	CM	Ketamine	1–100 mM	1 mM
[147]	Filter paper, 2-D	Wax printing	FLU	Au	0–750 nM	110 nM
[148]	Test card, 2-D	Wax printing	CM	Iodine	50–300 μg I/L	20 μg I/L
[150]	Filter paper, 2-D	Wax printing	FLU	Cu^2+^	5–1250 nM	5 nM
[152]	Filter paper, 2-D	Screen printing	EC	Dopamine Serotonin	30–800 μM 6–100 μM	0.13 μM 0.39 μM
[153]	Filter paper, 2-D	Cutting	FLU	Folic acid	1–300 μM	0.28 μM
[158]	Stripe paper, 3-D	Cutting + bonding	CM	Biomarker 1	30–50 ng/mL	5 ng/mL
[161]	Filter paper, 2-D	Immersed	CM	17β-estradio	0.1–1.0 μg/L	0.25 μg/L
[162]	Filter paper, 2-D	Wax printing	CM	Cysteine	0–1 mM	10 μM
[163]	Filter paper, 2-D	Wax printing	CM	Salbutamol	0.025–1 μg/L	0.025 μg/L

ATP: adenosine-50-triphosphate; CBD: Cannabidiol; CBN: Cannabinol; CM: Colorimetric; EC: Electrochemical; FLU: Fluorescence; ISE: selective electrode method; PS-MS: paper spray mass spectrometry; SERS: surface enhanced Raman spectroscopy.

## 4. Conclusions

Microfluidic lab-on-paper platforms are a rapidly developing and promising solution for the realization of next-generation preventive health care and detection analysis tools. Microfluidic lab-on-paper devices have many practical advantages over traditional laboratory systems, including a low cost, a simple manufacturing process, a straightforward operating procedure, good portability, high reliability, and a diagnostic performance close to that of benchtop methods. Furthermore, their use is compatible with many common biomedical samples, such as blood and urine. As a result, they have aroused great attention for POCT in the home or areas of the world with underdeveloped medical resources and infrastructures.

Urine has many advantages as a biomedical sample, including natural abundance, non-invasiveness and biological richness. Consequently, many microfluidic lab-on-paper platforms for urine analysis and disease detection have been proposed in recent years [100,108,164,165]. Many devices have also been successfully commercialized around the world, including the HiPee S2 smart urine analyzer (Tianjin Guo-Shih Tech. Co., Tianjin, China) and YH-1200 portable urine analyzer (Yao-Hua Co., Hebei, China). The MSLUA13 automatic urine analyzer (Medsinglong Medical Equipment Co., GuangZhou, China) has the ability to perform 14 different urine tests, including creatine, protein, microalbumin, specific gravity, calcium, nitrite, urobilinogen, pH, occult blood, glucose, bilirubin, ketone bodies, white blood cells, and vitamin C. Similarly, the Multistix 10 SG reagent strip device (Siemens Medical Solutions Inc., Malvern, PA, USA) can perform 10 urine tests, such as leukocytes, nitrite, urobilinogen, protein, pH, occult blood, specific gravity, ketone, bilirubin, and glucose. The test multi-drogues device produced by NarcoCheck Co. (Montluçon, France) provides the ability to test for 12 common drugs in urine, namely cannabis, cocaine, morphine, heroin, amphetamine, ecstasy, fentanyl, synthetic cannabinoids, ketamine, lysergic acid diacetamide, methylcystine and methamphetamine. Many simple urine test strips have also been commercialized for the detection of leukocytes and nitrites (Scanwell Health Co., San Diego, CA, USA), nicotine (Easy Healthcare Co., Willowbrook, IL, USA), pregnancy (Wondfo Biotech Co., GuangZhou, China) and ovulation (Femometer Co., Hong Kong, China). However, most of these devices provide only qualitative outcomes. Therefore, further research on the development of quantitative methods for the rapid analysis of urine samples using microfluidic lab-on-paper devices is still required.

The problem of improving the analysis accuracy of microfluidic lab-on-paper devices is also an ongoing concern. Studies have shown that the detection performance of colorimetric methods can be improved by inducing the aggregation of nanomaterials in order to enhance the detection resolution [119,149,156]. Similarly, in the EC and SERS methods, the detection performance can be improved by patterning nanomaterials with good electrical conductivity on the electrode surface [120,125,135,166,167]. In CL and fluorescence methods, the intensity of the detection signal can be amplified through the catalytic reaction of nanomaterials and the induced fluorescence quenching [132,168,169,170], respectively. Many studies have confirmed that the detection performance of microfluidic lab-on-paper platforms can be improved through the use of sample pre-concentration techniques (such as ICP, ITP, EKS, and so on) [72,73,75,115,171], or amplification devices (e.g., LAMP, PCR, CHR, HCR, and so forth.) [78,81,83,172,173]. For example, the authors in [84] showed that the detection performance of a lab-on-paper platform for urine microalbuminuria diagnosis in diabetic patients could be significantly improved by incorporating EKS with a 100-fold amplification capability in the sample pre-treatment stage.

Although lab-on-chip devices that use human urine samples for human disease diagnosis have achieved rapid development in recent years, there are still several key challenges that need to be resolved. For instance, due to the irregularity of the fiber structure of paper-based materials, achieving precise fluid control and quantitative sample transportation is extremely difficult, and leads to potentially serious reproducibility and stability issues. Accordingly, the development of porous materials with a uniform pore size distribution is essential in improving the reproducibility and detection sensitivity of future lab-on-paper devices. Recent studies have reported that glass fiber paper and nitrocellulose membranes have a uniform pore size distribution [5,10,16,17,18], and therefore offer an improved detection sensitivity and reproducibility. However, the manufacturing process for such materials is complicated and is more expensive than that for regular paper. In addition, although paper-based devices have many advantages over traditional benchtop systems for clinical diagnosis purposes, many of the sample pretreatment processes required to enhance the detection performance of these devices still require the use of large-scale laboratory equipment. As a result, further research is required to develop on-chip sample pretreatment methods in order to fully realize the commercial potential of paper-based microfluidic devices for POCT applications. Finally, there are still several remaining target analytes for detection by using urine samples and lab-on-paper platforms that have not been considered, including ketone body, Leu. esterase, epithelial cells, occult blood, and casts. In addition, lap-on-paper has insufficient research on virus infection or COVID in urine sample, mainly due to the detection limit. If lap-on-paper can be combined with amplification system [77] or immunosensor [174], it is research that can be developed. Hence, future studies are also needed to develop effective lab-on-paper platforms to detect such analytes.

In summary, this review describes all the key developments and concepts achieved in the microfluidic lab-on-paper platform field over the past five years for diagnosing and analyzing common human diseases with urine samples. It is expected that this review will help improve the availability and quality of medical services in developing areas of the world and accelerate the development of microfluidic lab-on-paper platforms in the field of human disease detection.

## Figures and Tables

**Table 1:** Comparison of detection using human samples.

Human Sample	Blood	Urine	Saliva	Sweat
Sample type	Invasive	Non-invasive	Non-invasive	Non-invasive
Sampling	Difficult	Easy	Easy	Easy
Sample volume	Lots	Lots	Little	Little
Sample pretreatment	Complicated	Easy	Easy	Easy
Detection accuracy	High	Medium	Medium	Medium
Detection cost	High	Low	Low	Low
Detectable items	Diversity	Diversity	Limited	Limited

## Data Availability

The data presented in this study are available upon request from the corresponding author.
